# Optimal power settings have major impact on procedural efficiency in pulmonary vein isolation guided by robotic magnetic navigation

**DOI:** 10.1007/s10840-025-02058-z

**Published:** 2025-06-04

**Authors:** Rita B. Gagyi, Ioan A. Minciuna, Tamas Geczy, Attila Nemes, Tamas Szili-Torok

**Affiliations:** 1 Cardiology Center, Department of Internal Medicine, University of Szeged, Szeged, Hungary; 2 5th Department of Internal Medicine, Faculty of Medicine, “Iuliu Hațieganu” University of Medicine and Pharmacy, Cluj-Napoca, Romania; 3 Cardiovascular Center, Bad Bevensen, Germany

**Keywords:** Pulmonary vein isolation, Robotic magnetic navigation, Catheter ablation

## Abstract

**Introduction:**

Early reports on pulmonary vein isolation (PVI) for atrial fibrillation (AF) guided by robotic magnetic navigation (RMN) show comparable results to manual radiofrequency (RF) or cryoballoon ablation; however, lengthy procedures were reported. This could be due to suboptimal lesion formation caused by a lack of user experience and defined best practices. Operators performed RMN-guided RF ablation with lower power settings, presumably leading to longer procedures. In this study, we aimed to re-evaluate safety and efficiency of RMN-guided PVI for AF based on delivered radiofrequency power.

**Methods:**

Patients undergoing RMN-guided ablation were screened, and consecutive patients with AF undergoing PVI-only between 2008 and 2023 were retrospectively enrolled. Patients were grouped by the power settings utilized during the PVI procedure (group 1—25-30W, group 2—30-35W, group 3—35-40W, group 4—40-45W, group 5—45-50W, and group 6—50-55W). We collected and analyzed demographic data: age, sex, and AF type; safety data: intra- and post-procedural complications; procedural data: procedure duration, fluoroscopy time, RF ablation time, RF application number, and success rate; and follow-up data: AF recurrence and number of redo procedures.

**Results:**

From the total number of 3398 screened patients, 238 patients met the inclusion criterion of undergoing PVI-only procedure (mean age 60.4 ± 9.9 years, 63.8% male). Throughout the 15 years only five patients had major (2.2%) and 15 patients had minor complications (6.6%), without differences between the patient groups (*p* = 0.40 and *p* = 0.63). The mean procedure duration was progressively decreased with the use of higher RF power (273.9 ± 97.0, 179.8 ± 104.0, 134.9 ± 55.3, 134.0 ± 39.5, 118.1 ± 41.3, and 110.9 ± 39.0 min, respectively; *p* < 0.001). Median fluoroscopy time was 19.5 min (IQR 13.0—35.5), progressively decreasing within the power groups (58.2 ± 20.5, 40.5 ± 26.2, 15.9 ± 6.6, 17.8 ± 8.1, 17.4 ± 7.5, and 19.8 ± 9.3 min; *p* < 0.001). We found differences between the power groups in RF application number (*p* < 0.001) and RF application duration (*p* = 0.003). Successful PVI was achieved in 238 patients (100.0%). Twenty-one patients with paroxysmal AF (17.1%) and 31 patients with persistent AF (40.7%) had documented recurrence during the 12-month follow-up. We found no differences in AF recurrence between the patient groups (*p* = 0.18 and 0.66).

**Conclusions:**

RMN-guided PVI-only for AF is safe and feasible. In contrast to early reports, procedure times and fluoroscopy use gradually decreased during the years, when increasing RF power was applied. Higher power settings during robotically-guided PVI did not compromise the safety of the procedures.

**Graphical Abstract:**

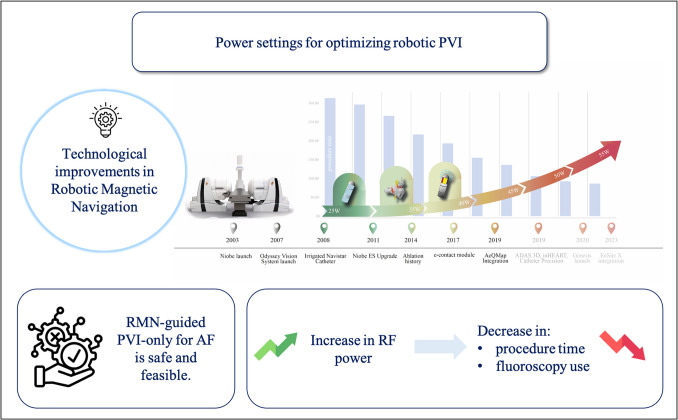

## Introduction

Robotic magnetic navigation (RMN) has a far-reaching history in cardiac catheter ablation (CA) procedures. Since being introduced in the early 2000 s, it has emerged as a potentially important tool to facilitate CA of atrial and ventricular arrhythmias [1, 2]. Recently, it has been proven markedly efficient in the treatment of complex arrhythmias [3, 4]. The myriad of studies assessing the added value of RMN suggests mostly either equal (in atrial arrhythmias) or superior efficacy (in ventricular arrhythmias) as compared to manual ablation procedures [5–10].

Despite recent scientific evidence, it has been a common misperception that the use of RMN is associated with longer procedure times in ablation of atrial fibrillation (AF). This is most likely triggered by early clinical data showing excessive procedure times during AF ablation [11, 12]. The possible reasons for this could be related to the following issues: at its early stage RMN did not have a sufficient catheter-tissue contact indicator (e-Contact Module—introduced in 2017) and radiofrequency energy delivery tracking (Ablation history module—introduced in 2014). Additionally, we presume that these initial results were influenced by the general belief among users that the constant catheter-tissue contact provided by RMN required lower power energy delivery to eliminate the risk of complications.

In the present study, we aimed to re-evaluate safety, efficiency, and efficacy of RMN-guided pulmonary vein isolation for AF based on the used power settings. The primary hypothesis of this study is that with progressively increased delivered radiofrequency (RF) power, procedural efficiency improves with unchanged clinical outcomes without compromising the well-documented safety profile of the robotic system [13].

## Material and methods

### Primary hypothesis and study design

The institutional medical ethics committee approved the data collection for this retrospective, single center study and concluded that it did not fall under the Medical Research Involving Human Subjects Act (SERCA and SERCA-2, MEC-2021–0299).

The primary endpoints of this study were safety characterized by intra- and post-procedural complications and procedural efficiency characterized by procedure duration. The secondary endpoints were fluoroscopy time, RF ablation time, number of RF applications, and efficacy characterized by success rate, AF/atrial tachycardia (AT) recurrence, and number of redo procedures.

**Fig. 1:**
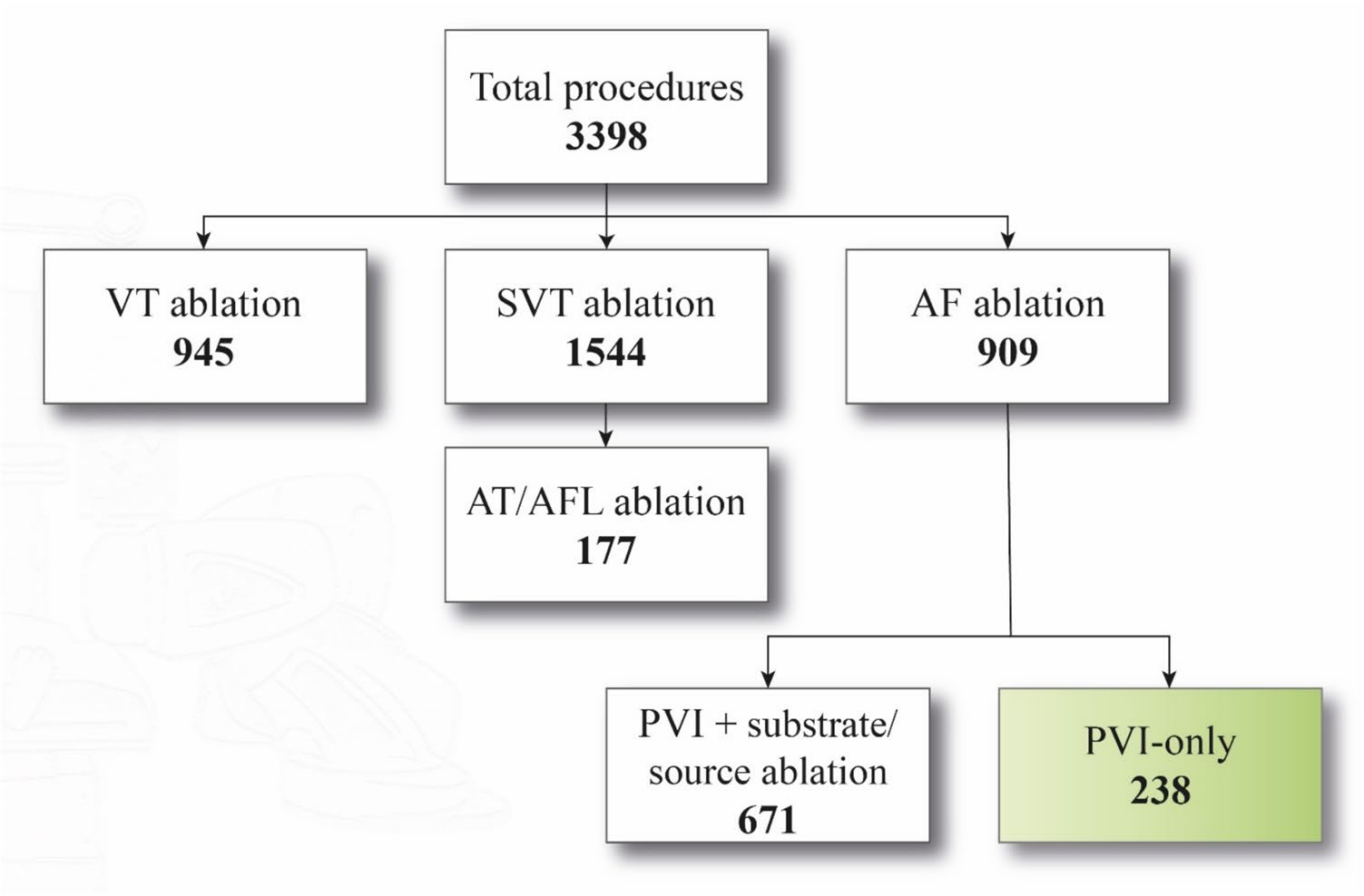
RMN-guided procedure summary: a total number of 3398 procedures from 2008 to 2023 were screened. In this study, we included 238 patients who underwent RMN-guided PVI-only procedure

This prospective registry screened all RMN-guided procedures and included patients with AF undergoing pulmonary vein isolation (PVI) only between 2008 and 2023 (Fig. 1). In order to avoid selection bias, we included all consecutive patients. Inclusion criteria were the diagnosis of AF and PVI-only procedure with the guidance of RMN (Stereotaxis Inc., St. Louis, MO, USA). Individuals who underwent repeat-PVI and/or additional linear or substrate ablation were excluded from the study. We grouped our patients based on power delivery settings, as follows: group 1—power setting 25–30 W group 2—30–35 W, group 3—35–40 W, group 4—40–45 W, group 5: 45–50 W, and group 6—50–55 W (posterior wall-anterior wall, respectively, in all cases). The level of delivered RF power progressively increased during the years, but it was possibly influenced also by the operator’s discretion.

Further, we included a subset of patients who underwent minimally invasive PVI-only procedures performed using the latest approach (MING—Minimally INvasive Group) without the inclusion of clinical fellows. All analysis was performed separately for the MING.

Due to the limited number of patients included in each study group, we performed a sub-analysis combining patient groups 1 + 2, 3 + 4, and 5 + 6. We analyzed follow-up data such as 12-month AF recurrence and overall atrial arrhythmia recurrence.

### Definitions

PVI-only procedures are defined as procedures where only PVI was performed during CA for the first time without additional line or substrate ablation. PVI was assessed by entrance and exit block pacing post-ablation. Major complications were defined as any procedure-related adverse events, which were life threatening, required significant surgical intervention, increased hospital admission time, led to disability or permanent damage, or resulted in death. Minor complications were defined as adverse events which resulted in minimal transient impairment of a body function or damage to a body structure, or which did not require any intervention. Puncture site complications included bleeding, hematoma formation and arteriovenous fistula formation, which required surgical intervention, prolonged hospitalization, and/or a Hb drop of > 1.8 mmol/L. Total procedure time was defined as the time passed from first venous puncture until the removal of sheaths. Acute success was defined as isolation of all PVs showing bidirectional conduction block.

Recurrence was defined as AF recorded on a 12-lead electrocardiogram (ECG) or AF lasting > 30 s on 24-h to 7-day continuous Holter monitoring. AT/atrial flutter (AFL) recurrence was defined as any AT/AFL documented on 12-lead ECG or 24-h to 7-day continuous Holter recordings, regardless of its duration.

### Data collection

Baseline demographic and clinical characteristics from patients were collected from our prospective database using electronic health records (HiX version 6.2, ChipSoft) and analyzed in accordance with hospital institutional review board policies. Procedural data were derived from the electronic medical files. The following demographic and procedural data were collected: sex, age, AF type, date of procedure, procedure duration (min), number of RF applications, total RF application duration (sec), power setting (watts), fluoroscopy time (min), acute success, hospital stay, and acute intra-procedural and post-procedural complications.

### PVI for paroxysmal AF

Pre-operatively, the presence of intra-cardiac thrombus was evaluated by trans-esophageal echocardiography (TEE). All procedures were performed under general anesthesia. The procedures started with (double) right femoral and (double) left femoral venous puncture to obtain venous access. Two 8 Fr sheaths were placed in the right femoral vein; one 6 Fr and one 10 Fr sheath were inserted in the left femoral vein. After a decapolar diagnostic catheter was positioned in the coronary sinus (CS), an ICE-guided septal puncture was performed to obtain transseptal LA access using either BRK1 needle, NRG RF Transseptal needle (Baylis Medical, Mississauga, ON, Canada), or, most recently, AcQCross transseptal access system (Acutus Medical Inc., Carlsbad, CA, USA) [14].

A circular Lasso catheter (Biosense Webster, Diamond Bar, CA, USA) and a 4-mm tip Navistar RMT ThermoCool ablation catheter (Biosense Webster, Diamond Bar, CA, USA) were advanced into the left atrium via a SL-1 long sheath (Abbott, Chicago, IL, USA). Passive recrossing was performed with an Agilis 8.5 Fr medium curl sheath (Abbott, Chicago, IL, USA). Electroanatomical mapping was performed using the CARTO 3 (Biosense-Webster Inc., Diamond Bar, CA, USA) mapping system by sweeping the Lasso catheter around the LA. After the LA map was created, all side branches of PVs were mapped in more detail using the ablation catheter. Once the mapping was complete, PVI was performed by a wide-area-circumferential-ablation (WACA) of all PVs. The WACAs were applied with continuous dragging of the ablation catheter while ablating with the following settings: maximum radiofrequency power 25–55 W, temperature limit 43 °C, and 17–30 mL/min irrigation. The duration of the catheter application at a single point was defined based on the combination of the ablation history values and the diminished electrograms. When the circular ablation of the PVs did not result in successful isolation, additional touch-up was performed until PV isolation. After successful isolation of both left- and right-sided PVs, a standard of 10- to 30-min waiting time was applied to identify early PV reconnection. We administered intravenous heparin for anticoagulation with a targeted ACT between 270 and 300 s. ECV was performed when indicated. Sheaths and catheters were removed when electrical isolation of all PVs was complete at the end of the waiting time. All procedures were performed in the same robotic operating room by the same team of highly experienced operators.

### Minimally invasive PVI strategy

Patients in the MING underwent a minimally invasive PVI-only procedure using the latest approach. Minimally invasive PVI procedures were performed as follows: the operator performed a single groin, double puncture to obtain vascular access. TEE-guided transseptal puncture and recrossing were performed to obtain double transseptal LA access. Anatomic CARTO mapping of the LA was performed sweeping the Lasso catheter around the chamber, all side branches of all PVs were mapped more in-detail using the ablation catheter. After completion of mapping, ablation of both left-sided PVs was performed until successful isolation, followed by isolation of both right-sided PVs, or vice versa. The WACA’s were applied using relatively high power settings (50–55 W, flow 17 mL/min, maximum 43 °C), with continuous dragging of the ablation catheter while ablating. Catheter positioning was constantly optimized using real-time feedback provided by the *Ablation History* and *e-Contact Module (ECM).* Additional touch-up was performed when necessary. After successful isolation of both left- and right-sided PVs, a standard of 10-min waiting time was applied to identify early PV reconnection.

### The RMN technology

The robotic system has been described in depth before [15, 16]. In short, two external permanent magnets located on either side of the patient generate a magnetic field, which can remotely direct the movement of the ablation catheter containing three magnets in the distal portion. The RMN system offers a so-called Ablation History, which provides a 3D visualization of the history of the catheter’s power output and RF application duration at each location on the CARTO 3 map during ablation. Based on the applied energy (W·s) at every location, targets are colored yellow (short application duration and/or low power applied) to orange (long application duration and/or high power applied) on the 3D CARTO screen. In order to determine catheter-tissue contact, the ECM was developed, which analyzes 16 different variables, including both unipolar and bipolar impedance measurements, and cardiac-induced catheter motion among others factors. In case of an optimal catheter-tissue contact a starburst indicator displays near the catheter tip and a blue line appears on the contact indication tracing [17].

### Follow-up

After each procedure, patients were monitored by 24-h telemetry. Before hospital discharge, regular access site checks, post-procedural echocardiography and ECG recordings were performed to screen for post-procedural complications. Antiarrhythmic medication was unchanged after the procedures and discontinued after 3 months if no recurrence was documented.

Routine follow-up visits were scheduled in the outpatient clinic of our department for all patients at 3, 6, 9, and 12 months after the procedures. Holter recordings from 24 h up to 7 days were employed during the follow-up visits for documentation of recurrent arrhythmias. We analyzed patient records for long-term follow-up.

### Statistical analysis

Mean and standard deviation (SD) were calculated for normally distributed continuous variables. Median and interquartile range (IQR) were computed for continuous variables with non-normal distribution. Descriptive statistics for categorical data were expressed in absolute numbers and percentages and were analyzed using the chi-square test or, when appropriate, Fisher’s exact test. Non-normally distributed data was analyzed using Kruskall-Wallis. Normally distributed continuous variables were analyzed using 1-way ANOVA. A two-sided *p*-value of < 0.05 was considered significant. The data was analyzed using SPSS 28.0 (SPSS Inc., Chicago, IL, USA). Arrhythmia-free survival was tested by the Kaplan–Meier survival curve with log-rank analysis.

## Results

### Demographic and baseline clinical data

**Fig. 2:**
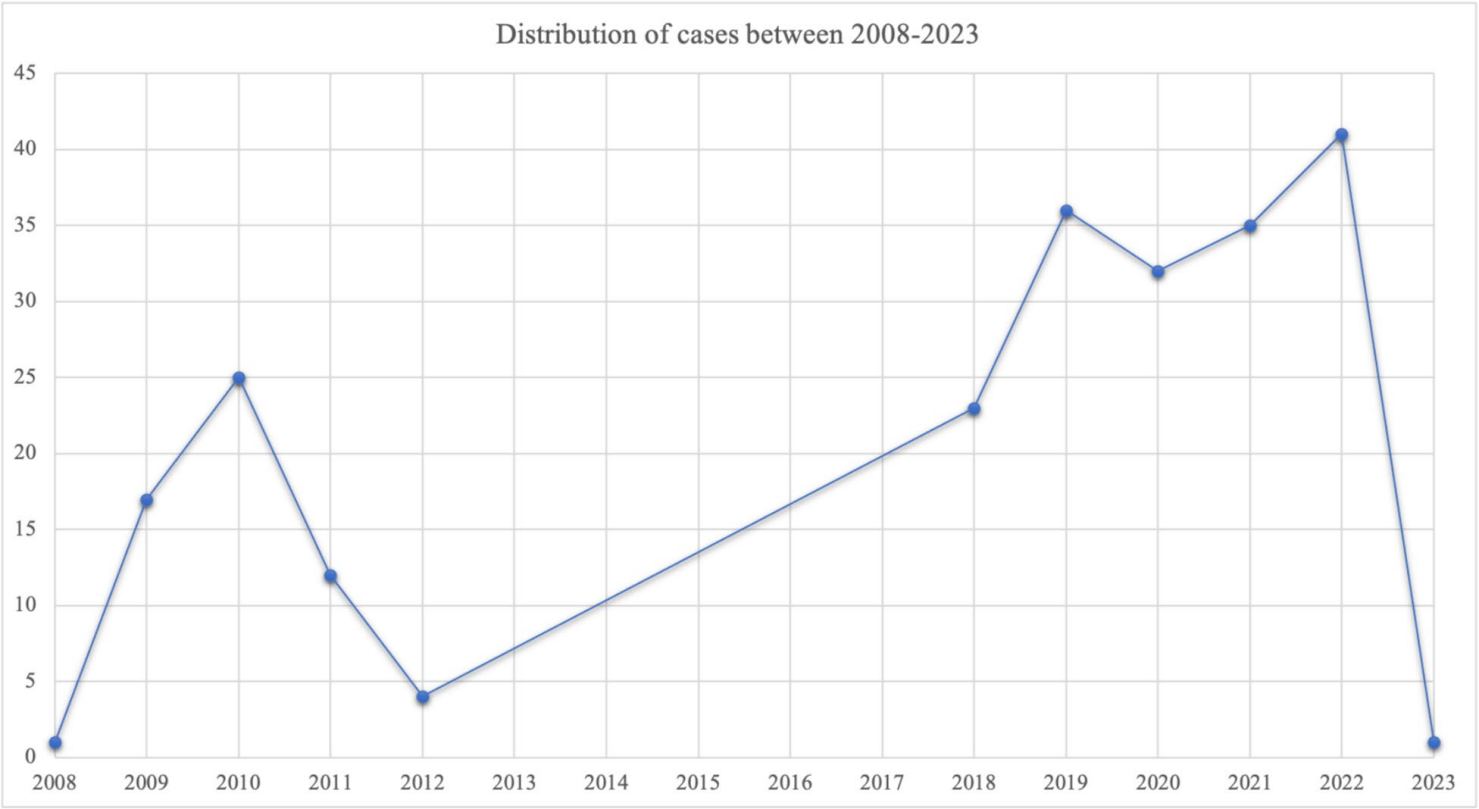
Distribution of cases during the years

A total of 3398 RMN-guided procedures were screened, and a total number of 238 patients were included in the final analysis, who underwent PVI-only using RMN (Fig. 1). The mean age of the included patients was 60.4 ± 9.9 years, and 63.8% of patients were male. Two hundred twenty-seven patients underwent de novo PVI, and 11 patients underwent minimally invasive PVI-only (MING). The following number of patients were included per year: 2008 = 1, 2009 = 17, 2010 = 25, 2011 = 12, 2012 = 4, 2018 = 23, 2019 = 36, 2020 = 32, 2021 = 35, 2022 = 41, and 2023 = 1 (Fig. 2). The following number of patients was included in the predefined power groups: power group 1 = 50 patients, power group 2 = 15, power group 3 = 9, power group 4 = 45, power group 5 = 93, and power group 6 = 15 patients. One hundred forty-seven patients had documented paroxysmal AF (64.8%), and 80 patients had persistent AF procedure (35.2%).

In the MING, the following patients were included per year: 2020 = 6, 2021 = 2, and 2022 = 3 patients. Eight patients were diagnosed with paroxysmal AF, 3 patients with persistent AF. All patients underwent minimally invasive de novo PVI-only procedure with the following power setting of 50–55 W.

### Safety data

**Fig. 3:**
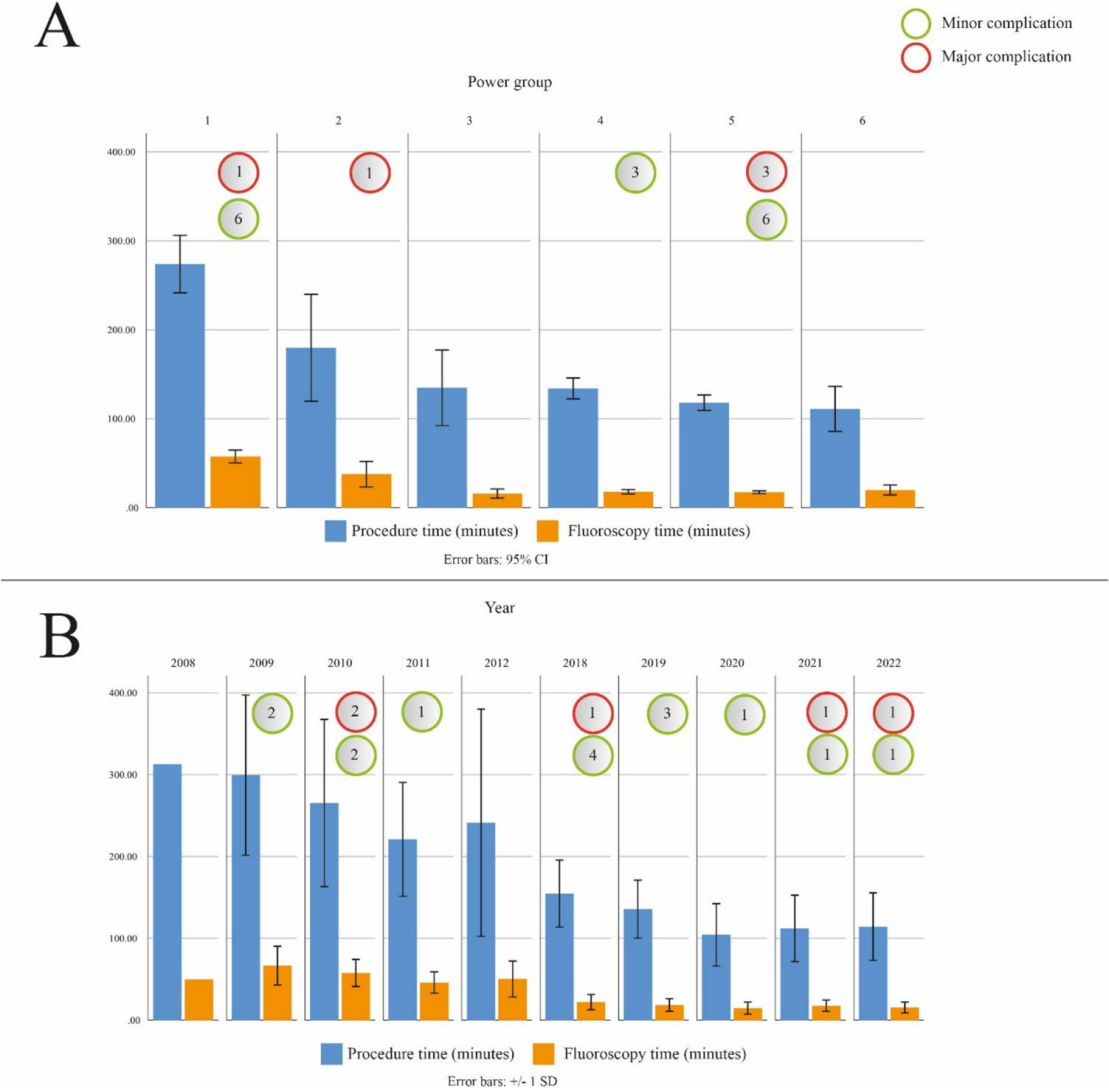
Safety and efficiency data: **A** procedure time and fluoroscopy time are presented by power group and **B** procedure time and fluoroscopy time are presented by year. Minor complications are illustrated with green circles; major complications are illustrated with red circles

Five patients had a major post-procedural complication (2.2%), including one patient with phrenic nerve paresis, one patient with pericarditis and lung embolism, 2 patients with TIA and one patient with CVA. There was no difference in major complication rate between power groups (1 in power group 1, 1 in power group 2, 0 in power group 3, 0 in power group 4, 3 in power group 5, and 0 in power group 6; *p* = 0.63). Two patients with major complications were discharged after 1 day of hospitalization, and 3 patients were hospitalized for 3 additional days (average hospitalization for patients with major complications 2.2 ± 0.9 days). Fifteen patients had a minor post-procedural complication (6.6%), including 9 patients with femoral hematoma, 5 patients with puncture site bleeding and one patient with back wound due to warming of the indifferent electrode. There was no difference in minor complication rate between the power groups (6 in power group 1, 0 in power group 2, 0 in power group 3, 3 in power group 4, 6 in power group 5, and 0 in power group 6; *p* = 0.40). A summary of complications per power group and per year is illustrated on Fig. 3.

In the MING, one patent suffered a minor procedure related complication (groin hematoma).

### Procedural efficiency

The mean procedure duration across all patient groups and all years was 153.5 ± 83.3 min, with significant differences between the power groups (273.9 ± 97.0 min in power group 1, 179.8 ± 104.0 in power group 2, 134.9 ± 55.3 in power group 3, 134.0 ± 39.5 in power group 4, 118.1 ± 41.3 in power group 5, and 110.9 ± 39.0 min in power group 6; *p* < 0.001) and between years (313.0 min in 2008, 299.4 ± 97.9 in 2009, 265.4 ± 102.1 in 2010, 221.0 ± 69.5 in 2011, 241.3 ± 138.9 in 2012, 154.7 ± 41.0 in 2018, 135.7 ± 35.4 in 2019, 104.3 ± 38.1 in 2020, 112.2 ± 40.7 in 2021, and 114.1 ± 40.3 min in 2022; *p* < 0.001). Median fluoroscopy time across all groups was 19.5 (IQR 13.0–35.5) min, progressively decreasing within the power groups (58.2 ± 20.5 min in power group 1, 40.5 ± 26.2 in power group 2, 15.9 ± 6.6 in power group 3, 17.8 ± 8.1 in power group 4, 17.4 ± 7.5 in power group 5, and 19.8 ± 9.3 min in power group 6; *p* < 0.01) and between years (50.0 min in 2008, 66.3 ± 22.9 in 2009, 60.6 ± 16.6 in 2010, 44.6 ± 13.1 in 2011, 50.3 ± 21.9 in 2012, 22.1 ± 9.2 in 2018, 18.6 ± 7.6 in 2019, 14.7 ± 7.2 in 2020, 17.7 ± 6.9 in 2021, and 15.2 ± 6.6 min in 2022; *p* < 0.01). Data of procedure duration and fluoroscopy time in more detail are illustrated on Fig. 3. We found difference between the power groups in RF application number (93.5 ± 43.3 in power group 1, 78.5 ± 63.2 in power group 2, 27.7 ± 12.6 in power group 3, 25.7 ± 13.3 in power group 4, 23.5 ± 17.3 in power group 5, and 21.8 ± 9.5 in power group 6; *p* < 0.01), RF application duration (2349.6 ± 1160.9 s in power group 1, 2508.1 ± 1258.5 in power group 2, 2364.4 ± 1059.7 in power group 3, 2008.7 ± 675.9 in power group 4, 1874.3 ± 737.3 in power group 5, and 1565.4 ± 388.2 in power group 6; *p* = 0.003) and hospital stay (3.5 ± 1.5 days in power group 1, 2.9 ± 1.5 in power group 2, 1.5 ± 0.5 in power group 3, 1.6 ± 0.6 in power group 4, 1.4 ± 0.5 in power group 5, and 1.3 ± 0.4 in power group 6; *p* < 0.01). Procedural data by power group is summarized in Table 1. Procedural data by year is summarized in Table 2.

**Table 1:** Procedural data by power group

	Power group						*p-* value
	1	2	3	4	5	6	
Patient numbers	50	15	9	45	93	15	
Procedure time (min) ± SD	273.9 ± 97.0	179.8 ± 104.0	134.9 ± 55.3	134.0 ± 39.5	118.1 ± 41.3	110.9 ± 39.0	< 0.001
Application number ± SD	93.5 ± 43.3	78.5 ± 63.2	27.7 ± 12.6	25.7 ± 13.3	23.5 ± 17.3	21.8 ± 9.5	< 0.001
Application time (seconds) ± SD	2349.6 ± 11609	2508.1 ± 1258.5	2364.4 ± 1059.7	2008.7 ± 675.9	1874.3 ± 737.3	1565.4 ± 388.2	0.003
Fluoroscopy time (min) ± SD	58.2 ± 20.5	40.5 ± 26.2	15.9 ± 6.6	17.8 ± 8.1	17.4 ± 7.5	19.8 ± 9.3	< 0.001
Hospital stay (days) ± SD	3.5 ± 1.5	2.9 ± 1.5	1.5 ± 0.5	1.6 ± 0.6	1.4 ± 0.5	1.3 ± 0.4	< 0.001

**Table 2:** Procedural data by year

	Years									p-value
	2009	2010	2011	2012	2018	2019	2020	2021	2022	
Patient numbers	17	25	12	4	23	36	32	35	41	
Procedure time (min) ± SD	299.4 ± 97.9	265.4 ± 102.1	221.0 ± 69.5	241.3 ± 138.9	154.7 ± 41.0	135.7 ± 35.4	104.3 ± 38.1	112.2 ± 40.7	114.1 ± 40.3	< 0.001
Application number ± SD	94.5 ± 26.4	115.5 ± 60.5	70.0 ± 23.4	63.0 ± 0.0	23.1 ± 16.5	19.6 ± 12.8	26.4 ± 12.3	27.3 ± 19.8	27.3 ± 15.6	< 0.001
Application time (seconds) ± SD	2505.1 ± 691.4	2837.3 ± 1613.4	1942.5 ± 619.6	1299.7 ± 1118.3	2132.2 ± 889.5	1951.5 ± 823.5	1761.3 ± 542.2	1905.7 ± 734.8	1912.1 ± 653.1	< 0.001
Fluoroscopy time (min) ± SD	66.3 ± 22.9	60.6 ± 16.6	44.6 ± 13.1	50.3 ± 21.9	22.1 ± 9.2	18.6 ± 7.6	14.7 ± 7.2	17.7 ± 6.9	15.2 ± 6.6	< 0.001

One hundred fifteen procedures were performed by a single operator (50.7%) and 112 procedures with an electrophysiology fellow (49.3%).

**Fig. 4:**
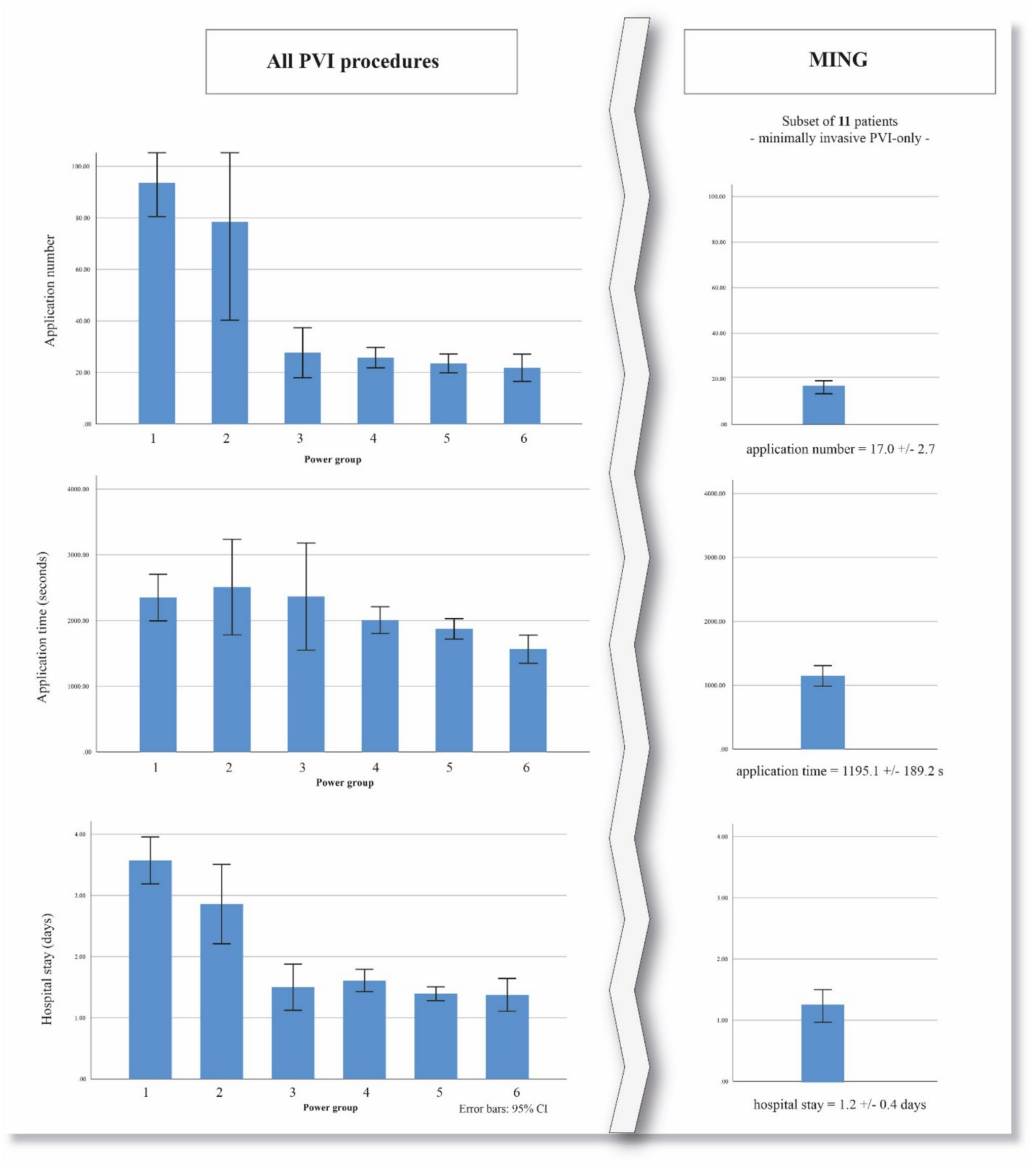
Application number, duration, and hospital stay: on the left, application number, duration, and hospital stay are summarized by power group. On the right, all these variables are summarized for the subset of MING patients

In the MING, mean procedure duration was 63.3 ± 11.6 min, with a fluoroscopy time of 11.6 ± 3.9 min. The average number of RF application was 17.0 ± 2.7 and average application duration 1195.2 ± 189.2 s. Average hospital stay was 1.2 ± 0.4 days. Visual comparison of the subset of patients with power groups is shown in Fig. 4.

### Efficacy data

Successful PVI was achieved in 238 patients (100.0%). Fifty-two patients had AF recurrence within 12 months (from 199 patients where 12-month follow-up data was available) (26.1%).

### Paroxysmal AF recurrence rates

**Fig. 5:**
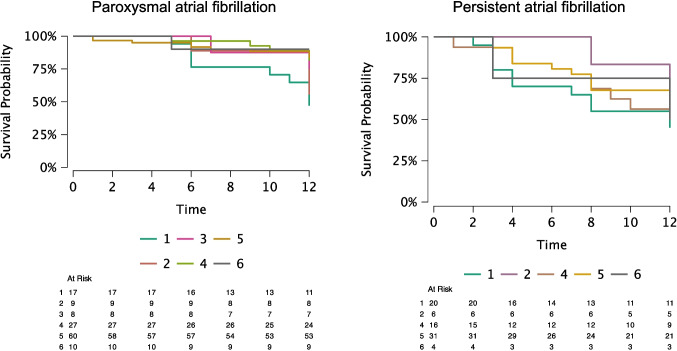
Arrhythmia-free survival analysis for paroxysmal and persistent AF stratified by power group

Twenty-one patients with paroxysmal AF had AF recurrence within 12 months (out of 123, 17.1%), and there were no differences between the power groups: 7 patients in power group 1 (41.2%), 1 patient in power group 2 (12.5%), 2 patients in power group 3 (25.0%), 3 patients in power group 4 (11.5%), 7 patients in power group 5 (12.0%), and 1 patient in power group 6 (16.7%) (*p* = 0.18). For patients with paroxysmal AF, 31 had AF recurrence after 12 months (27.2%), with average time to recurrence of 16.7 months. In total, 32 patients with paroxysmal AF underwent a redo procedure for AF either within or after 12 months from the initial PVI (32/134, 23.9%). There were no differences in redo procedures between the power groups (*p* = 0.55). Seventeen patients with paroxysmal AF had AT recurrence within 12-months (13.8%), and 5 patients underwent a second EP procedure for AT ablation (4.1%). Freedom from all atrial arrhythmias was 47.0% in group 1, 55.5% in group 2, 75.0% in group 3, 81.4% in group 4, 83.3% in group 5, and 90.0% in group 6 (*p* = 0.02). Arrhythmia-free survival is shown on Fig. 5.

In the sub-analysis there was no difference in 12-month AF recurrence between the combined groups (groups 1 + 2: 32.0%, groups 3 + 4: 14.2%, and groups 5 + 6: 12.3%, *p* = 0.21). When analyzing overall atrial arrhythmia recurrence, we found 50.0% recurrence in groups 1 + 2, 20.0% recurrence in groups 3 + 4, and 15.7% recurrence in groups 5 + 6 (*p* < 0.01).

### Persistent AF recurrence rates

Thirty-one patients with persistent AF had documented recurrence within 12 months (31/76, 40.7%). Ten patients in power group 1 (50.0%), 2 patients in power group 2 (40.0%), one patient in power group 3 (100%), 7 patients in power group 4 (43.8%), 10 patients in power group 5 (32.3%), and 1 patient in power group 6 (33.3%) (*p* = 0.66). A total of 33 patients with persistent AF had documented AF recurrence after 12 months (33/72, 45.8%), with an average time to recurrence of 13.0 months. Twenty-nine patients with persistent AF underwent a redo procedure for AF either within or after 12-months from the initial PVI (29/80, 38.2%). There were no differences in redo procedure between the power groups (*p* = 0.57). Eight patients with persistent AF had AT recurrences during the 12-month follow-up (8/78, 10.3%) and 3 patients underwent a redo procedure for AT (3/78, 3.7%). Freedom from all arrhythmias was 45.0% in group 1, 50.0% in group 2, 100.0% in group 3, 50.0% in group 4, 64.5% in group 5 and 50,0% in group 6 (*p* = 0.64). Arrhythmia-free survival is shown on Fig. 5.

In the sub-analysis there was no difference in 12-month AF recurrence between the combined groups (groups 1 + 2: 48.0%, groups 3 + 4: 47.0%, and groups 5 + 6: 31.4%, *p* = 0.37). When analyzing overall atrial arrhythmia recurrence, we found 53.8% recurrence in groups 1 + 2, 52.9% recurrence in groups 3 + 4, and 37.1% recurrence in groups 5 + 6 (*p* = 0.35).

### Recurrence rates in the Minimally INvasive Group

In the MING, 2 patients with paroxysmal AF had documented AF recurrence and 1 patient had documented AT recurrence during the 12-month follow-up. Two patients with persistent AF had documented AF recurrence during the 12-month follow-up. In total, 4 patients continued to present with AF recurrence after 12 months from the minimally invasive PVI-only procedure (3 patients with paroxysmal and 1 patient with persistent AF). Two patients with paroxysmal AF underwent a redo procedure during or after the 12-month follow-up period.

## Discussion

The major finding of this study is that with progressively increasing delivered RF power, procedure duration using RMN decreases substantially, and outcomes of RMN-guided procedures remain effective without compromising the excellent safety profile of the robotic system. When applying higher power setting, overall arrhythmia recurrence rates are lower after paroxysmal atrial fibrillation ablation guided by RMN.

### Early results of robotic AF ablation

In the existing literature, early reports on PVI for AF guided by RMN show comparable results to manual or cryoballoon ablation, however procedure times often exceed 3 h [18–20]. In these reports, while the effectiveness of RMN demonstrated to be optimal, experts considered long procedure times a potential major disadvantage. According to the authors, the following factors might have contributed to excessive procedure times: increased number and duration of RF lesions and difficulty in achieving the end-point [16, 20].

In 2007, Di Biase et al. reported on a study including 45 consecutive patients who underwent circumferential PVI guided by the earliest version of RMN. During the procedures, they used a relatively high-power setting of 40–50 W, and they observed charring on the non-irrigated ablation catheter tip in 33% of patients. While they were performing a point-by-point PVI with coordinate and wand approach, they could achieve PV disconnection in only 4 patients [16]. Based on these discouraging early results, users concluded that effective lesion sets for RMN-guided PVI could not be achieved with the present catheter technology [16]. In the following years, several studies were conducted assessing the safety and feasibility of RMN-guided AF ablation, using second-generation ablation catheters but with significantly lower RF power settings between 20 and 35 W. In contrast to the study published by Di Biase et al., these studies showed improved efficiency; however, long procedure times remained a major limitation [12, 20–22].

The results of the present study validate these early reports. We found that in the first years of implementation of RMN in our center, most procedures exceeded 3 h, a successful PVI required around 80–90 RF applications, and application durations varied between 2000 and 2500 s (30–40 min). 

### Misperception of constant contact

It seems possible that the above-described initial results played an important role in a more permanent misperception of the catheter-tissue contact capability of the RMN system. Before the introduction of contact force sensing catheters in 2012, tactile feedback estimation was considered as a standard way to estimate the contact between the catheter tip and cardiac tissue. Obviously, tactile feedback was not an option for remotely guided procedures and even though the RMN system was designed to offer constant catheter-tissue contact provided by its magnetic field, in the lack of a reliable contact indicator users applied RF with lower power settings. By applying low RF power, lesions set were suboptimal, multiple ablation sets were necessary, and the chances of reaching the end-point of a standard PVI in reasonable time decreased.

It was only later, in 2018, when Bessière et al. demonstrated on an experimental model that the magnetic field created by the RMN system provides constant and stable catheter contact forces with an average of 6 g without a sheath (and 20 g with long sheath) [23]. In their study, they provide justification to use higher power settings, considering that the average contact force is somewhat lower compared to standard manual ablation procedures [23]. The highest contact force they were able to achieve using RMN was 22 g, which is a reassuring safety feature supporting current reports on the absence of cardiac perforation as a major complication. The present study supports evidence from these previous observations by proving that while power settings were increased, the number of major complications did not change. Nevertheless, while experienced RMN users recommended applying higher power settings than for standard manual procedures, there is not one existing protocol or guideline on the recommended power settings for RMN-guided PVI. During every robotic PVI procedure, power settings are adjusted at the discretion of the operator. As we demonstrate in the present study, when lacking a predefined power setting protocol, the efficiency of RMN-guided procedures is highly operator-dependent. Our results show that the shortest procedure and fluoroscopy times and the lowest RF application numbers and durations were documented during PVI procedures performed with the latest minimally invasive approach (MING) (Fig. 4).

### Technological improvements

**Fig. 6:**
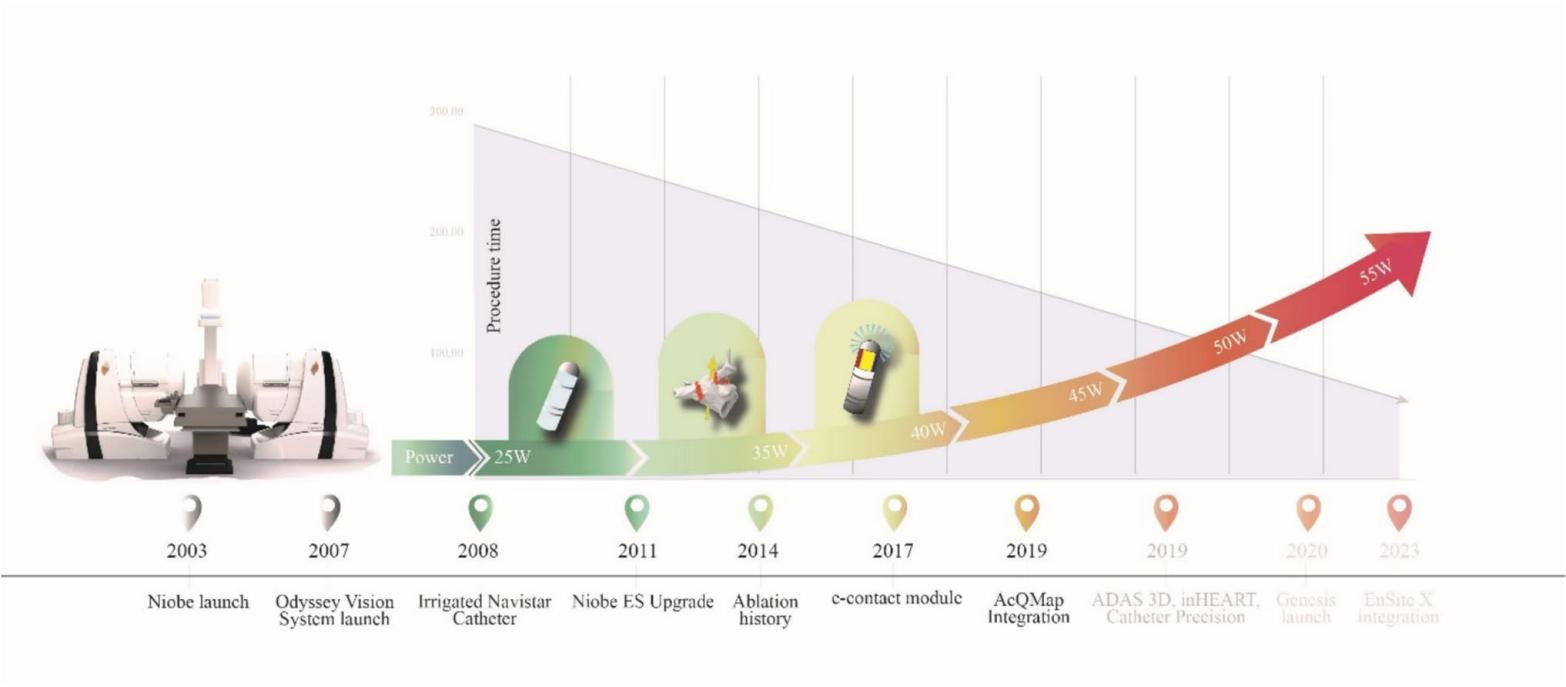
Technological improvements

Since the introduction of the Niobe RMN system in 2003, it has gradually evolved into a sophisticated platform with numerous advantages for CA of various arrhythmias. Introduced in 2007, the first version of the Odyssey Vision system aggregates and integrates a vast amount of clinical information (such as ultrasound, ECG, and 3D mapping) during an EP procedure into one large screen with single mouse & keyboard control of all systems. This allows the operator to focus on a consolidated view when controlling various systems employed in the procedure. An irrigated diagnostic and ablation Navistar RMT catheter was introduced in 2008, overcoming the limitations of the previously used ablation catheters and providing a safe option for ablation without charring or coagulation formation. Introduced in 2011, the Epoch platform was a comprehensive upgrade for the Niobe system and was geared to improve efficiency of CA procedures and increase workflow. The Epoch solution was expected to considerably cut procedure times for all types of robotically guided procedures, including PVI. The Ablation History feature was introduced later, in 2014, and provides a history of the catheter's power output and duration of energy application at each location during the ablation through an intuitive graphical display. With this advantageous feature, the operator can retrace ablation-related activity and potentially identify gaps in lesion sets on a real-time basis. To overcome critics for the inability to titrate contact force, Stereotaxis introduced the novel catheter-tissue contact feedback feature, the ECM, in 2017. With this reliable contact feedback, the operator can optimize catheter position before applying RF energy, thus preventing inadequate lesion formation. A next big step towards an optimized and personalized approach was the integration of the novel dipole charge density mapping system, AcQMap (Acutus Medical Inc., Carlsbad, CA, USA) in 2019, followed by the integration of the EnSite X (Abbott, Chicago, IL, USA) in 2023. Combining the precise navigation capabilities of the RMN with high-resolution 3D mapping systems allows the operators to visualize and navigate complex cardiac anatomies with unparalleled accuracy. All the above-described developments combined create a new and better environment for robotically guided CA. Technological improvements are illustrated on Fig. 6.

### Recent results

In the last 5 years, a series of studies demonstrated that with the technical advances made in the RMN ablation technique, it has become equally efficient as manual or cryoballoon ablation in performing PVI for AF. In a study published in 2019 by van den Bruck et al., authors using the old Niobe II version of the RMN system achieve inferior efficiency results compared to manual procedures [24]. They report an average procedure duration of 265 min for 80 patients, highlighting the lack of operator experience and low number of procedures as study limitations. In the same year, in a study published by Noten et al., authors assess efficiency parameters in manual, cryoballoon and RMN-guided PVI procedures, comparing a group of patients treated with the old Niobe II version and a group of patients treated with the next generation Niobe ES. Their results demonstrate that the greatest magnitude of procedure time improvement was observed within the RMN groups, with the new version of Niobe ES operators achieved an average procedure time of 113 min. Regarding first-pass isolation, the highest first-pass isolation rates were observed in the robotic magnetic navigation group (78% and 74%; left and right PVs respectively), which was significantly higher compared to manual (24% and 24%); and cryoballoon procedures (50% and 48%; P < 0.001) [6]. In the present study, we do not report PV reconnection rates; however, the inclusion of such data would significantly enhance the comprehensiveness of the study and enable the derivation of broader conclusions. Li et al. reported an average procedure time of 142.5 min for PVI or redo PVI in 502 patients with AF ablated using RMN after 2015; however the authors do not report on power settings [25]. These results validate the advantages offered by technological developments implemented in the past years. This also accords with our observations illustrated in Fig. 3, which shows that procedure and fluoroscopy times gradually decreased from 2008 to present.

A more recent study published by Noten et al. in 2022 describes a minimally invasive ablation strategy using the RMN system for PVI in 14 patients with a power setting of 45–50 W. In their paper, the authors describe the latest available approach for PVI-only procedures, achieving a mean procedure time of 63 min with a mean fluoroscopy time of 12 min [26]. The results of the present study correspond to the above-described outcomes. We found that procedure times and fluoroscopy times gradually decreased by increasing the applied RF power, and average procedure time in the MING group was 63.3 min, with a fluoroscopy time of 11.6 min. Although not statistically significant, our data shows a trend toward improvement in efficacy with decreasing percentage of AF recurrence during 12-month follow-up. The suboptimal long-term outcomes in persistent AF ablation raise the question whether PVI-only is the ultimate solution for persistent AF patients, or if we should aim for a more personalized approach. With recently published studies showing constantly improving efficiency data of AF ablation and reporting on higher RF power settings, it is likely that previous RMN-guided ablations were underpowered, the lack of operator experience influenced efficiency, or the combination of both apply.

### Limitations

The major limitation of this study is that it is a non-randomized single-center study. However, this longitudinal study includes a relatively high number of patients, and randomization for this patient population would not have been feasible. In the early years of RMN use in our center, data on first-pass isolation rate was not available. Upon reviewing our data, it is evident that the distribution of patients across the various power groups is unequal, which poses a significant limitation from a statistical perspective. However, as previously noted, these groups accurately reflect the real-world evolution of applied power settings over the years. Consequently, we conducted a pertinent sub-group analysis to demonstrate that this limitation does not impact the outcomes of the study. Our analysis concluded that the increased use of RF power does not adversely affect the outcomes of the procedures.

## Data Availability

The data that support the findings of this study are available from the corresponding author (T. Sz-T.) upon reasonable request.

## Abbreviations

CA: Catheter ablation
AF: Atrial fibrillation
PV: Pulmonary vein
PVI: Pulmonary vein isolation
TEE: Trans-esophageal echocardiography
AFL: Atrial flutter
ECG: Electrocardiogram
RF: Radiofrequency
ECV: Electrical cardioversion
CS: Coronary sinus
CVA: Cerebrovascular accident
ACT: Activated clotting time
LA: Left atrium
RA: Right atrium
LSPV: Left superior pulmonary vein
LIPV: Left inferior pulmonary vein
RSPV: Right superior pulmonary vein
RIPV: Right inferior pulmonary vein
SVC: Superior vena cava
LAA: Left atrial appendage
IQR: Interquartile range
SD: Standard deviation
